# Optimising diagnostics for hard-of-hearing infants: factors associated with successful MRI scanning without general anaesthesia

**DOI:** 10.1007/s00405-024-09118-6

**Published:** 2025-01-17

**Authors:** Marlise D. van der Veen, Ithri Kaman, Bas Jasperse, Thadé Goderie, Fenna A. Ebbens, K. Mariam Slot, Marjo S. van der Knaap, Paul Merkus

**Affiliations:** 1 Otolaryngology – Head and Neck Surgery, section Ear & Hearing, Amsterdam UMC, Vrije Universiteit Amsterdam, De Boelelaan 1117, Amsterdam, 1081 HV The Netherlands; 2 Radiology and Nuclear Medicine, Amsterdam UMC, Vrije Universiteit Amsterdam, De Boelelaan 1117, Amsterdam, 1081 HV The Netherlands; 3 Neurosurgery, Amsterdam UMC, Vrije Universiteit Amsterdam, De Boelelaan 1117, Amsterdam, 1081 HV The Netherlands; 4 Paediatric Neurology, Amsterdam UMC, Vrije Universiteit Amsterdam, De Boelelaan 1117, Amsterdam, 1081 HV The Netherlands; 5 Amsterdam Public Health Research Institute, Quality of Care, Amsterdam, The Netherlands; 6 Amsterdam Neuroscience Research Institute, Cellular & Molecular Mechanisms, Amsterdam, The Netherlands

**Keywords:** Paediatrics, Hearing loss, Feed-and-swaddle, Feed-and-sleep, Magnetic resonance imaging, Paediatric anaesthesia

## Abstract

**Purpose:**

Scanning during infancy is often required in otology, preferably without general anaesthesia. This study aims to determine the success rate of MRI of the head without general anaesthesia for infants, and to identify predictors for a successful scan.

**Methods:**

Data was extracted from the electronic patient file for patients who received MRI of the head without general anaesthesia between 01-01-2019 and 31-12-2022 at an age younger than 6 months. Each MRI-session was dichotomised into success (i.e., of sufficient quality to answer the clinical question) or failure, and success percentages were calculated. A logistic regression analysis was performed to determine the association between success and variables of interest, which were selected based on interviews with medical specialists.

**Results:**

Eighty-seven patients were included, showing an overall success rate of 75.9% for MRI of the head without anaesthesia. Success rates for MRI brain were higher than for MRI cerebellopontine angle (CPA), respectively 91.2% and 66.0% (*p* = 0.013). For MRI CPA the odds of success decreased for infants aged 3–5 months, compared to infants under 3 months (respectively 48.1% and 84.6%, *p* = 0.009). For MRI CPA the success percentage was lower for boys (51.9%) than for girls (80.8%, *p* = 0.039). Time of day and hearing loss showed no significant effect on the success rate.

**Conclusion:**

Obtaining MRI of the head without anaesthesia for infants under six months is feasible. For MRI CPA the success rate is higher for infants scanned at a younger age, as well as for female infants compared to male infants.

## Introduction

When requesting Magnetic Resonance Imaging (MRI) for an infant, it can be difficult to determine whether the infant is able to undergo the scan in their natural sleep or whether general anaesthesia is required for successful imaging. For physicians requesting MRI scans of the head for infants, it is important to make a good prediction of the chance of success for imaging without anaesthesia as using anaesthesia has several downsides, but failed scans should be avoided as well. Therefore, the goal of this study is to determine the success rate of MRI scanning of the head without anaesthesia in infants under 6 months, and to find factors that are associated with the prediction of this success rate.

## Background

**Fig. 1:**
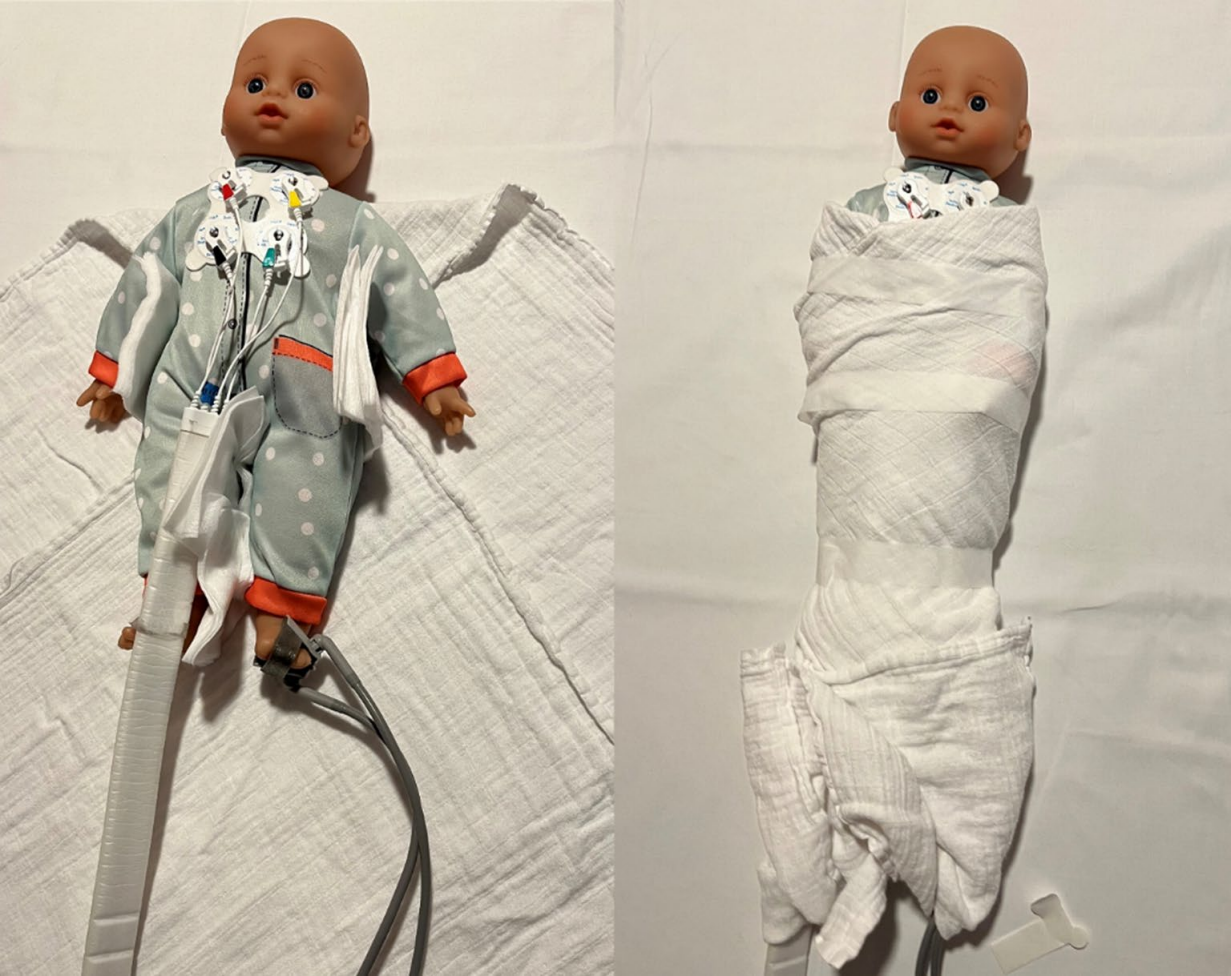
An impression of the swaddle technique, which is applied prior to scanning, to obtain an MRI scan of infants without general anaesthesia. The infant is first fed by one of the parents or caregivers. After feeding, an MRI technician applies equipment to monitor heart rate and blood pressure throughout the scan. Pieces of cloth are placed in between the legs and arms, to prevent skin from touching. The MRI technician then tightly swaddles the infant using (thin) blankets. This will encourage the child to fall asleep and remain still during the scan. Earplugs, a headphone or cushions can be used to protect from the loud scanning noises

Magnetic Resonance Imaging is an imaging modality commonly used in otology, to identify anatomic abnormalities associated with hearing loss, or prior to surgery, such as cochlear implantation [1]. Since MRI is a radiation-free imaging modality providing superior contrast of soft tissues, such as the brain, and the cochlear and vestibular nerve, it is often the modality of choice for imaging children [2, 3]. However, for obtaining an MRI scan the patient has to lie still for a relatively long time, as examinations consist of multiple sequences, each often lasting several minutes [4]. General anaesthesia is therefore frequently required for young children who are yet unable to follow instructions [5, 6]. This puts an extra burden on the patient, requires the deployment of an anaesthesiologist, and increases the cost of the procedure. Next to these factors, there has been a growing concern that exposure to general anaesthesia under the age of 3 years is associated with long-term cognitive deficits [7, 8]. Therefore, it is preferred to scan young infants without general anaesthesia, but in their natural sleep, using a so-called feed-and-sleep or feed-and-swaddle technique [9]. Figure 1 gives an impression of the swaddle technique as used in our centre.

In our centre there are no formal eligibility criteria for using the feed-and-swaddle procedure, so the decision to scan either with general anaesthesia or using the feed-and-swaddle technique is based on the assessment by the ENT-doctor requesting the scan, in consultation with the parents or caregivers of the infant. To avoid failed scans due to motion artefacts and to provide parents or caregivers with reasonable expectations it is important that the physician requesting the scan has a good estimate and prediction of the chance of success for MRI without anaesthesia. Various studies have described the feed-and-swaddle technique and have proven its feasibility [10, 11]. However, those studies were not aimed at *predicting* the success of MRI without anaesthesia or were not focused on scans of the head, which might be more sensitive to motion artefacts [12–15]. A pilot study with 26 patient files was aimed at finding predictors of a successful MRI in natural sleep, but for most potential predictors, the sample size was insufficient to find a statistically significant effect [16].

Our study aims to determine the success rate of MRI scanning of the head without general anaesthesia in children under 6 months, and to identify factors associated with predicting the chance of success.

## Methods

### Study cohort & inclusion criteria

For this retrospective review, records of patients from the departments of otorhinolaryngology, neurosurgery and paediatric neurology of our single-centre tertiary care hospital were extracted based on the following inclusion criteria:

- An MRI was obtained for the patient between 01-01-2019 and 31-12-2022.
- Patients were younger than 6 months old at time of scanning.

Patients were excluded from the analysis if the scan was imported from another hospital, or if the scan was obtained using general anaesthesia.

This study and the applied no-objection procedure were approved by the hospital’s Medical Ethical Review Committee (study number: 2022.0417). Following the no-objection procedure, parents or caregivers of children eligible for inclusion received a letter informing them about the study and allowing them to object to inclusion of the patient’s data.

### Feed-and-swaddle and MRI procedure

To obtain MRI scans of infants without general anaesthesia a feed-and-swaddle procedure is followed. This procedure consists of feeding the infant immediately prior to scanning and swaddling the infant using a piece of cloth. In some cases, the child is given a few droplets of sucrose water during the scan and sequences might be repeated to obtain an image of sufficient quality. All scans included in this study were made using one of the MRI scanners available in our hospital, which are either Siemens Healthineers, Philips Healthcare, or GE Healthcare scanners, and 1.5 or 3.0 Tesla.

### Variables of interest

Potentially relevant variables were identified during interviews with physicians. Interviews were conducted with four ENT-surgeons and one paediatric neurologist who frequently request MRI scans of the head for infants, and three radiologists who regularly evaluate these scans. Various patient factors were hypothesised to be of relevance for the chance of successful scanning without general anaesthesia, but only age of the patient was mentioned to be frequently considered in the decision-making process. Potentially relevant variables were then selected for inclusion in the analysis based on availability of information on the variables in the Electronic Patient File. This led to the inclusion of five potentially relevant input variables: type of MRI scan, (corrected) age at time of scanning, sex of the patient, hearing loss, and time of day. The type of MRI scan was categorised into two groups: MRI brain or MRI cerebellopontine angle (CPA). The category MRI CPA contains MRI of the cerebellopontine angle as well as two MRI scans of the facial structures. Age at time of scanning was included in the database in months. In the case of prematurity (born at 37 weeks of pregnancy or less) the corrected age was used. The corrected age was calculated by subtracting the number of weeks the child was born prematurely from the given age, based on a standard of 38 weeks pregnancy [17]. Sex was included as a dichotomous variable (male or female). Hearing loss was categorised into three categories: no or unilateral hearing loss, mild to severe bilateral hearing loss, and profound bilateral hearing loss. The last category was defined as the patient being eligible for cochlear implantation (CI). In the Netherlands, patients with unilateral hearing loss are currently not eligible for CI. Lastly, the variable time of day was divided into morning (before 11:59 a.m.) and afternoon (after 12:00 p.m.).

### Outcome measure: success of the scan

The outcome measure in this study was success of the MRI scan, which was dichotomised into success or failure. Whether the scan was successfully obtained was determined based on the information on the quality of the scan in the radiologist’s report. A scan was considered successful if the quality was sufficient for a radiologic report and to answer the clinical question. If there were too many motion artefacts to answer the clinical question, or if a large part of the requested sequences were not performed, a scan was considered unsuccessful. Scans with some motion artefacts, a few sequences missing, or with repeated sequences were still considered successful if the radiologist deemed the scan suitable to make a statement about the clinical question.

### Statistical analysis

Success percentages were calculated for all independent variables. Univariable logistic regression analyses were performed to assess the association of the variables of interest with the odds of success. For the MRI CPA scans, a logistic regression model was created using a forward selection procedure to determine which variables to include. The sample size of our database allows for inclusion of two independent variables in the regression model. A conditional forward selection approach was used, with *p* < 0.05 set as condition for inclusion and *p* < 0.10 set as condition for removal from the model. Statistical significance was set at *p* < 0.05 and all statistical analyses were performed using SPSS version 28.0 (IBM Corp, Armonk, NY, USA).

## Results

### Demographics

Data was extracted for 139 MRI scans of the head obtained at an age below 6 months. Thirty-three scans were excluded because they were obtained using general anaesthesia. The majority of these were brain scans (*N* = 29) and most scans made using general anaesthesia were obtained at a corrected age of 4 (*N* = 11) or 5 (*N* = 14) months. An additional 15 scans had been imported from another hospital and were excluded as well. After following the no objection procedure, 87 scans were included in the analysis. The majority of scans were obtained at an age of 2 or 3 months, making up respectively 36.8% and 32.2% of all scans. An overview of demographics and the variables of interest is presented in Table 1.

**Table 1:** Patient demographics and occurrence of the variables of interest (*N* = 87)

		Total	MRI CPA ^a^	MRI brain
		N (%)	N (%)	N (%)
All		87 (100)	53 (60.9)	34 (39.1)
Age ^b^	0	1 (1.1)	1 (1.9)	0 (0)
	1	9 (10.3)	4 (7.5)	5 (14.7)
	2	32 (36.8)	21 (39.6)	11 (32.4)
	3	28 (32.2)	19 (35.8)	9 (26.5)
	4	13 (14.9)	6 (11.3)	7 (20.6)
	5	4 (4.6)	2 (3.8)	2 (5.9)
Sex	Male	46 (52.9)	27 (50.9)	19 (55.9)
	Female	41 (47.1)	26 (49.1)	15 (44.1)
Hearing loss ^c^	No/unilateral	50 (56.8)	15 (28.3)	35 (100)
	Mild-severe bilateral	21 (23.9)	21 (39.6)	0 (0)
	Profound bilateral	17 (19.3)	17 (32.1)	0 (0)
Time of day ^d^	Morning	40 (46.0)	22 (41.5)	18 (52.9)
	Afternoon	47 (54.0)	31 (58.5)	16 (47.1)

^a^ CPA indicates Cerebellopontine angle

^b^ Age in months, corrected for prematurity

^c^ Profound bilateral hearing loss is here defined as eligible for cochlear implantation

^d^ Time of day was classified as morning (up to 11.59 a.m.) or afternoon (from 12:00 p.m.)

### Success percentages

The overall success percentage of MRI scanning of the head without anaesthesia for all infants under 6 months included in this study was 75.9%. For MRI CPA the success percentage was 66.0%, which was lower than for MRI brain (91.2%, *p* = 0.013). To assess the association of the other variables with success of the scan, a separate analysis was performed for only the MRI CPA scans. For MRI brain only 3 scans in the study database were considered failed, which was insufficient to perform a separate statistical analysis for MRI of the brain. The failed MRI brain scans were obtained at an age of 1 month (*N* = 1) or 2 months (*N* = 2). One of the infants for whom a scan was unsuccessfully obtained was described to have difficulty with feeding.

### Linearity assumption for age

For age to be included as a continuous variable, logistic regression analysis requires a linear relationship between the logarithm of the odds ratio of successful scanning, and age. To assess this linearity assumption, age at time of scanning was divided into four categories (0–1 months, 2 months, 3 months, and 4–5 months). The success percentage in the first category (0–1 month) was 100%, so the odds ratio could not be calculated. This means the linearity assumption required for including age as a continuous variable in the logistic regression analysis, could not be checked. For further analysis, age at time of scanning was therefore dichotomised into 0–2 months and 3–5 months. The value of 3 months was based on the interviews, in which multiple physicians had mentioned an age of 3 months as a cut-off for the success of MRI without anaesthesia.

### Factors associated with success of MRI CPA

The success percentages of MRI CPA per independent variable and the corresponding p-values for univariable logistic regression are given in Table 2.

**Table 2:** Success of obtaining MRI CPA for infants under 6 months without general anaesthesia (*N* = 53), for various variables

		Successful scans (%)	Significance
All		35 (66)	
Age ^a^	0–2 months	22 (84.6)	
	3–5 months	13 (48.1)	0.008*
Sex	Male	14 (51.9)	
	Female	21 (80.8)	0.031*
Hearing loss ^b^	No/unilateral	11 (73.3)	
	Mild - severe bilateral	11 (52.4)	0.209
	Profound bilateral	13 (76.5)	0.838
Time of day ^c^	Morning	17 (77.3)	
	Afternoon	18 (58.1)	0.151

^a^ corrected for prematurity

^b^ No/unilateral hearing loss is set as reference category. Profound bilateral hearing loss is here defined as eligible for cochlear implantation

^c^ Time of day was classified as morning (up to 11:59 a.m.) or afternoon (from 12:00 p.m.)

*A significant effect (5%-level) was found for the variables age and sex

The results show a decrease in the odds of success for children aged 3 to 5 months (48.1% success), compared to children of 2 months or younger (84.6% success, *p* = 0.008). Female patients were more often successfully scanned than male patients, with success percentages of 80.8% and 51.9% respectively (*p* = 0.031). For hearing loss and time of day no significant association with the odds of a successful scan was found.

### Regression model for MRI CPA

**Fig. 2:**
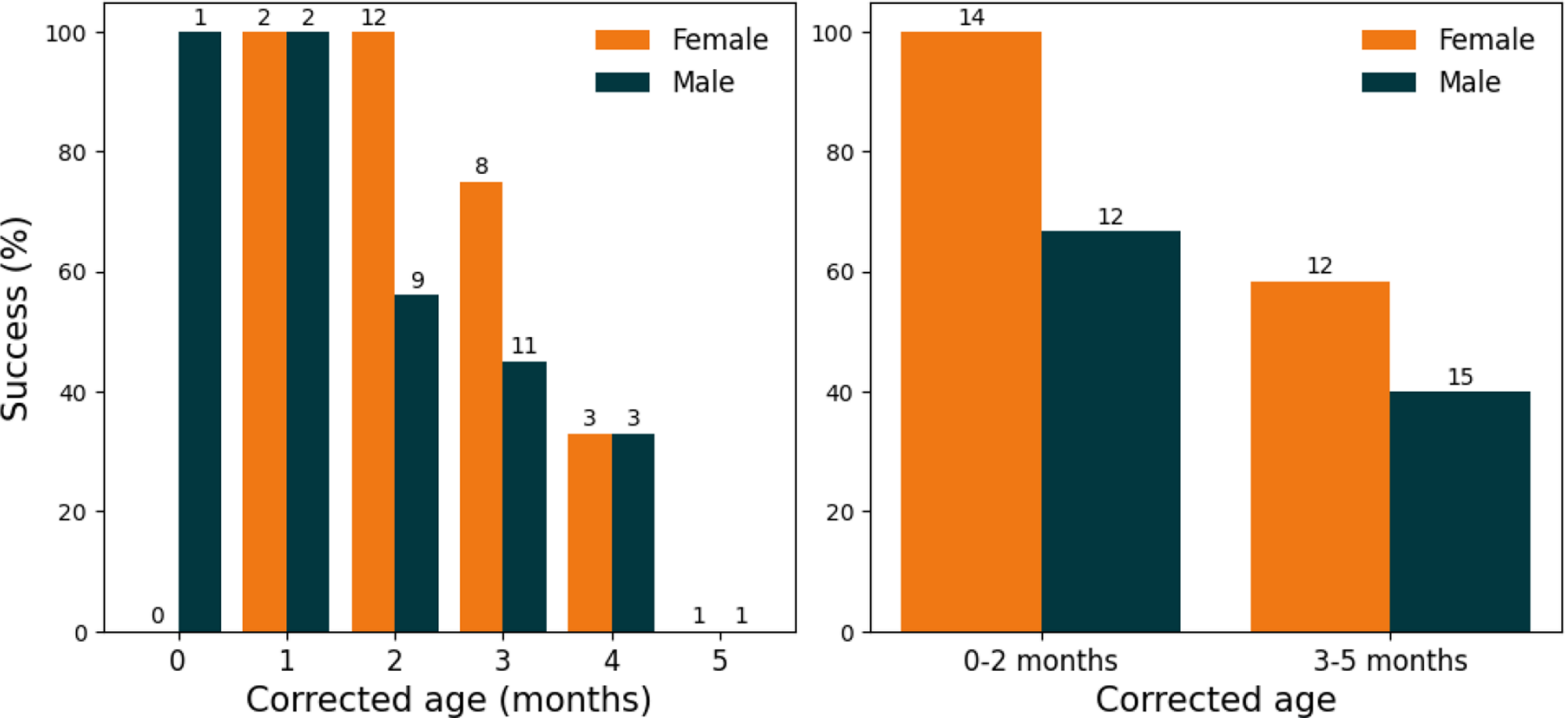
Success rates of MRI CPA without general anaesthesia for children under 6 months dependent of (corrected) age, shown separately for female and male patients. The total number of patients in each group is indicated above the bars

In Fig. 2, the success percentages of MRI CPA are shown separately for female and male patients, both per month and divided into the two age groups. The success percentage for female patients of 0 months (first 30 days after birth) is missing, since there were no female patients scanned at that age.

A prediction model for successful MRI scanning of the CPA was created with multivariable logistic regression, using a forward selection approach. The final model contained the dichotomous variables corrected age at time of scanning and sex as independent variables. The regression coefficients, odds ratios and significance levels are given in Table 3.

**Table 3:** Coefficients and significance levels in the logistic regression model for success of obtaining MRI CPA without general anaesthesia in children under 6 months

	Regression coefficient B	Odds Ratio (exp(B)) [95% confidence interval]	Significance
Age (3–5 months)	-1.819	0.162 [0.041;0.640]	0.009*
Sex (male)	-1.411	0.244 [0.064;0.929]	0.039*
Constant	2.526	12.505	< 0.001

^a^ corrected for prematurity

*Statistically significant at 5-percent level (*p* < 0.05)

To assess the quality of the prediction model, the area under the curve of the Receiver Operator Characteristic was calculated. The resulting value of 0.774, indicates acceptable performance of the model [18]. The expected success percentages and odds ratios per group, calculated using the regression coefficients, are given in Table 4.

**Table 4:** Expected success percentages of MRI CPA in infants based on age (corrected for prematurity) and sex of the infant, calculated using the regression coefficients. Corresponding expected odds ratios are given as well (in brackets)

	Female	Male
0–2 months	93% (12.5)	75% (3.05)
3–5 months	67% (2.03)	33% (0.49)

## Discussion

In this retrospective study we have demonstrated the feasibility of successfully obtaining MRI scans of the head of infants without general anaesthesia using the feed-and-swaddle technique, with an overall success rate of 75.9%. We have found that for MRI brain very few scans (3/34) were unsuccessful. For MRI CPA scans the success rate was higher for younger infants, and for female patients compared to male patients. The other variables (hearing loss and time of day) did not show an association with the success rate of MRI CPA.

### Success rates compared to literature

Various studies have already described the feed-and-swaddle technique for obtaining MRI scans of infants, with success rates for children ranging from 52% up to 100% [10–16, 19–22].

The reported success rates are in general higher than what we found in our study, which can largely be attributed to the type of MRI under investigation, or the age of the patients included in the studies. Fogel et al. [19], Shariat et al. [21] and Windram et al. [22] have reported success rates of 96–100%, but those studies focused specifically on cardiac or cardiovascular MRI, which might be less sensitive to motion artefacts. Success rates of 100% and 89% were reported by Golan et al. [20] and Hansen et al. [11], respectively. However, these studies did not focus on a specific type of MRI. Furthermore, in the study of Hansen et al. the majority of patients was younger than 1 week, with only a few patients aged over 12 weeks. Success rates from 79% (completely addressed the clinical question) up to 99% (at least partly addressed the clinical question) were described by Antonov et al. [12] for all types of MRI scans, but they have only included patients younger than 92 days, with the majority of included patients scanned at an age of less than 1 month. Moreover, only 1 patient was referred from the department of ENT. For full-body MRI without general anaesthesia a success rate of 94% has been reported by Gale et al. [10]. Although this was an extensive study, involving over 700 patients, most of the patients included were under 1 month of age, whereas approximately half of the patients in our study were aged 3 months or older.

Our results indicate that success rates of MRI scans of the head, particularly of MRI CPA for diagnostic purposes, cannot be directly adopted from success rates for other types of MRI scans. Imaging of anatomical structures related to hearing loss requires a high accuracy, making the scans sensitive to motion artefacts and thereby decreasing the chance of a successful scan. This assumption is supported by the difference we found in success rates between the MRI CPA scans and the MRI brain scans.

Four previous studies have investigated the feed-and-swaddle technique specifically for MRI scans related to sensorineural hearing loss (SNHL) [13–16]. Weng et al. only included patients with a corrected age of less than 13 weeks and found a success rate of 86.8% [13]. A similar success percentage (86%) was reported by Grose et al. for a relatively small patient population of 21 infants, of whom 14 were younger than 12 weeks [14]. A success percentage of only 52% was found by Liao et al., for patients with a median age of 3.2 months [16]. Ronner et al. reported a success rate of 82% for patients with a median age of 2.11 months [15].

The success rates found in our study are in general slightly lower than those presented in previous studies. When taking into consideration the type of scan and age of the infants, our results are in line with existing literature, but they provide more realistic expectations for an ENT clinic aiming to implement the feed-and-swaddle technique.

### Factors associated with success

Literature addressing factors influencing the chance of a successful MRI scan is limited. We found that the type of requested scan is an important predictor for success. This might be explained by differences in required accuracy for different scans and purposes.

Another possible explanation is a difference in scan duration or noise level of the scan. Unfortunately, information on duration or noise level of specific sequences is not available, neither within our hospital database, nor in existing literature.

Specifically for MRI CPA we found that scanning at a younger age is associated with a higher chance of success. Such an effect has also been reported by Weng et al. [13]. For scans related to hearing loss, Ronner et al. and Liao et al. did not find an effect of age on success chance [15, 16], but this is possibly due to small sample sizes. Liao et al. do imply a negative effect of increasing age on success chance might be present and become significant with a larger sample size.

The effect of sex on successful scanning has not been reported in previous literature. Liao et al. did find an odds ratio of 2.05 for successful scanning of female infants compared to male infants, but they could not prove the significance of this effect.

Two factors that have been reported to reduce the success rate of scanning without general anaesthesia are prematurity and comorbidity [12, 16]. Due to the presence of only 1 premature patient in our database and the wide variety of possible comorbidities, these factors were not considered as feasible separate independent variables in our study. We were therefore unable to verify the associations between prematurity and comorbidity with chance of success.

A final factor possibly related to successful scanning is hearing loss. Infants who are less able to hear the noise of the MRI scanner, are expected to be more likely to successfully undergo an MRI without general anaesthesia. Ronner et al. indeed found an increase in success rate for infants with bilateral profound SNHL, compared to infants with other types of hearing loss. However, when comparing bilateral SNHL with unilateral SNHL, they did not find a significant effect [15]. The results from Grose et al. suggest a link between bilateral hearing loss and an increased success rate compared to unilateral hearing loss, but they did not report a statistical test [14]. A positive, but insignificant relation between the severity of hearing loss and the chance of success was also mentioned by Liao et al. [16]. Our results show the success rates for patients with profound bilateral hearing loss are slightly higher than those for patients with mild to severe bilateral hearing loss as well, but this difference was not significant. This difference cannot be seen when comparing profound bilateral hearing loss to no/unilateral hearing loss. We are therefore unable to support the assumption that (profound) bilateral hearing loss leads to an increased chance of a successful scan. Possibly, other factors surrounding the MRI, such as temperature of the scanning room or movements of the scanning table, cause these infants to wake up, despite being less able or unable to hear the scanning noises. Further research with larger sample sizes might be able to reveal an association between hearing loss and successful scanning without general anaesthesia. However, based on our results we expect that an effect of hearing loss, if it exists, would be too small to be of clinical relevance for individual patients.

### Strengths

In this study we have provided proof of the feasibility of MRI scanning of the head without general anaesthesia. Additionally, we identified patient factors associated with the chance of success. There is very little information available on success factors of the feed-and-swaddle technique for MRI in general and even fewer studies have specifically investigated MRI of the head. Our results give clinicians requesting MRI scans for infants the opportunity to discuss the chances of a successful scan without general anaesthesia during consultation with parents or caregivers, to give a more personal estimate of the success chance and enable shared decision making. Our results can also be used to formulate a policy for scanning infants using the feed-and-swaddle technique, which optimizes the number of MRI scans made without general anaesthesia, while minimizing the number of failed attempts. Such a policy could consist of the advice to scan as early as possible, at least during the first three months of life, as this increases the chance of a successful scan.

Another strength of our study, next to the clinical applicability, is our relatively large sample size, compared to other studies that specifically investigated success of feed-and-swaddle MRI for infants with hearing loss [13–16].

### Limitations

Although our sample size is relatively large compared to many previous studies, considering the many factors potentially influencing the success of the scan, the sample size would preferably be even larger. A larger sample size would potentially allow for identifying additional relevant factors, such as hearing loss. It would also allow for a more precise evaluation of the effect of age on success by categorising age into smaller categories.

Since this is a retrospective study, the observed success rates might have been influenced by prior assessments of the physicians referring for a scan. Although our centre does not use formal eligibility criteria for applying the feed-and-swaddle technique, multiple physicians have mentioned they are more inclined to attempt scanning without general anaesthesia if the infant is younger. Our data indeed shows that for older infants, scans have frequently been obtained using anaesthesia, especially for MRI of the brain. Presuming the same age-relation observed for MRI CPA, applies to MRI brain, we expect that if all these patients had undergone MRI without anaesthesia, the overall success rates would have been lower.

Another limitation related to the availability of data is the possible influence of external factors on the odds of success. During the interviews, multiple physicians mentioned that tranquillity and patience are key in successfully obtaining an MRI of children. This means the odds of success might also be affected by external factors, such as patience of the MRI technician, time allocated for repeating sequences, noisiness of the waiting area, and presence of a parent in the scanning room. Such factors are not saved in the patient file and have therefore not been included. However, since all scans were made within the same centre, it is expected that these external factors have been similar for most patients, rendering it unlikely that the associations found in this research should actually be attributed to external factors.

A final limitation of this study is the uncertainty in the age of infants. The age at time of scanning has been corrected for prematurity based on a standard of 38 weeks pregnancy, but this correction was only applied for children born prematurely. For children born a term only the actual age was considered. Since full term babies are born at 37 up to 41 weeks pregnancy and pregnancy duration is not saved in the patient file, there is an uncertainty of approximately one month in the corrected age of infants. It should be noted that previous studies have shown an association between sex of the foetus and duration of pregnancy, where male foetuses have been associated with an increase in preterm births as well as prolonged pregnancies [23, 24]. This means that taking into account the corrected age compared to a standard of 38 weeks pregnancy for all patients, both premature and full or late term, might explain the difference between male and female patients found in our study.

### Clinical implications

This study shows that using the feed-and-swaddle procedure to obtain an MRI of infants under 6 months is feasible, making it a suitable alternative to scanning under general anaesthesia. When deciding to use the feed-and-swaddle method, a referring physician should consider the type of scan, since the success rate for MRI CPA is significantly lower than for MRI brain. Specifically for MRI CPA it is important to consider the age of the patient. The chance of success for younger infants (under 3 months) is higher than for older infants (3–5 months). It might be advisable to only scan infants under 3 months using the feed-and-swaddle procedure and to use general anaesthesia for older infants, especially if scanning capacity is an issue or rapid scanning results are of great importance. Our results can also be used in shared decision-making with parents or caregivers. An example of this could be to discuss with caregivers that the chance of a failed MRI CPA scan is higher for boys, especially if they are 3 months or older.

## Conclusions

The overall success rate for MRI of the head without anaesthesia for infants under 6 months was 75.9%. The success rate was lower for MRI CPA than for MRI brain. For MRI CPA a positive association was found between scanning at a younger age and the chance of a successful MRI. We found the success rate of MRI CPA to be higher for female patients than for male patients. These results can be used to form a policy on the use of the feed-and-swaddle technique as an alternative to scanning with general anaesthesia, for infants referred by ENT doctors.

## Data Availability

The data supporting the findings of this study are not publicly available due to privacy restrictions. Upon reasonable request from relevant individuals an anonymised data set can be shared by the corresponding author.
