# Correction to: Optimising diagnostics for hard-of-hearing infants: factors associated with successful MRI scanning without general anaesthesia

**DOI:** 10.1007/s00405-025-09308-w

**Published:** 2025-04-02

**Authors:** Marlise D. van der Veen, Ithri Kaman, Bas Jasperse, Thadé Goderie, Fenna A. Ebbens, K. Mariam Slot, Marjo S. van der Knaap, Paul Merkus

**Affiliations:** 1 Otolaryngology– Head and Neck Surgery, Section Ear & Hearing, Amsterdam UMC, Vrije Universiteit Amsterdam, De Boelelaan 1117, Amsterdam, 1081 HV The Netherlands; 2 Radiology and Nuclear Medicine, Amsterdam UMC, Vrije Universiteit Amsterdam, De Boelelaan 1117, Amsterdam, 1081 HV The Netherlands; 3 Neurosurgery, Amsterdam UMC, Vrije Universiteit Amsterdam, De Boelelaan 1117, Amsterdam, 1081 HV The Netherlands; 4 Paediatric Neurology, Amsterdam UMC, Vrije Universiteit Amsterdam, De Boelelaan 1117, Amsterdam, 1081 HV The Netherlands; 5 Amsterdam Public Health Research Institute, Quality of Care, Amsterdam, The Netherlands; 6 Amsterdam Neuroscience Research Institute, Cellular & Molecular Mechanisms, Amsterdam, The Netherlands

**Correction to: European Archives of Oto-Rhino-Laryngology**

10.1007/s00405-024-09118-6

In this article, few values were incorrectly published and it should have been as in this correction.

1. ***In the results section of the Abstract***, the sentence ***“***For MRI CPA the odds of success decreased for infants aged 3–5 months, compared to infants under 2 months (respectively 84.6% and 48.1%, *p* = 0.009).” should have been “For MRI CPA the odds of success decreased for infants aged 3–5 months, compared to infants under 3 months (respectively 48.1% and 84.6%, *p* = 0.009).”
2. ***In the Results section***,*** under “Factors associated with success of MRI CPA***”, the sentences ***“***The results show a decrease in the odds of success for children aged 3 to 5 months (48.1% success), compared to children of 2 months or younger (81.5% success, *p* = 0.010). Female patients were more often successfully scanned than male patients, with success percentages of 80.8% and 50% respectively (*p* = 0.018).” should have been “The results show a decrease in the odds of success for children aged 3 to 5 months (48.1% success), compared to children of 2 months or younger (84.6% success, *p* = 0.008). Female patients were more often successfully scanned than male patients, with success percentages of 80.8% and 51.9% respectively (*p* = 0.031).”
3. ***In the Discussion section***, the sentence ***“***In this retrospective study we have demonstrated the feasibility of successfully obtaining MRI scans of the head of infants without general anaesthesia using the feed-and-swaddle technique, with an overall success rate of 76.1%.” should have been “In this retrospective study we have demonstrated the feasibility of successfully obtaining MRI scans of the head of infants without general anaesthesia using the feed-and-swaddle technique, with an overall success rate of 75.9%.”
4. ***In the Conclusion section***, the sentence ***“***The overall success rate for MRI of the head without anaesthesia for infants under 6 months was 76.1%.” should have been “The overall success rate for MRI of the head without anaesthesia for infants under 6 months was 75.9%.”

The original article has been corrected.

